# Small Bowel Obstruction due to Mesodiverticular Band of Meckel's Diverticulum: A Case Report

**DOI:** 10.1155/2010/901456

**Published:** 2010-08-09

**Authors:** Aziz Sumer, Ozgur Kemik, Aydemir Olmez, A. Cumhur Dulger, Ismail Hasirci, Umit Iliklerden, Erol Kisli, Cetin Kotan

**Affiliations:** ^1^Department of Surgery, Faculty of Medicine, Yuzuncu Yıl University, 65080 Van, Turkey; ^2^Departments of Gastroenterology, Faculty of Medicine, Yuzuncu Yıl University, 65080 Van, Turkey

## Abstract

Meckel's diverticulum is the most common congenital anomaly of the small intestine. Common complications related to a Meckel's diverticulum include haemorrhage, intestinal obstruction, and inflammation. Small bowel obstruction due to mesodiverticular band of Meckel's diverticulum is a rare complication. Herein, we report the diagnosis and management of a small bowel obstruction occurring due to mesodiverticular band of a Meckel's diverticulum.

## 1. Introduction

Meckel's diverticulum is the most common congenital anomaly of the gastrointestinal system [1–3]. It originates from failure of the vitelline duct to obliterate completely, which is usually located on the antimesenteric border of the ileum. Its incidence is between 1% and 3% [2, 4, 5]. Meckel's diverticulum occurs with equal frequency in both sexes, but symptoms from complications are more common in male patients. Most of the Meckel's diverticula are discovered incidentally during a surgical procedure performed for other reasons. Haemorrhage, small bowel obstruction, and diverticulitis are the most frequent complications. Histologically, heterotopic gastric and pancreatic mucosa are frequently observed in the diverticula of symptomatic patients [1, 3]. Involvement of the mesodiverticular band of the diverticulum is seen rarely. 

Herein, we were able to clearly demonstrate the mesodiverticular band. We reported the diagnosis and management of a small bowel obstruction due to mesodiverticular band of a Meckel's diverticulum.

## 2. Case Report

**Figure 1:**
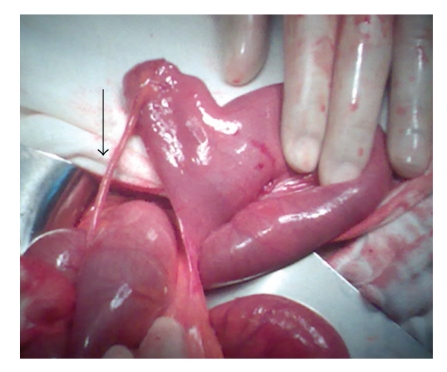
Meckel's diverticulum and mesodiverticular band (arrow).

**Figure 2:**
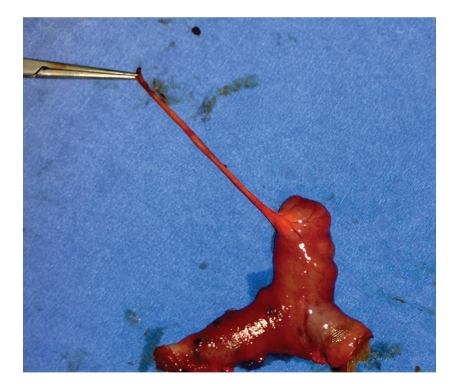
The view of resected Meckel's diverticulum and mesodiverticular band.

A 17-year-old male with no previous abdominal surgery, who experienced severe abdominal pain and vomiting one day earlier, was admitted to the emergency service of our hospital. His abdomen was very tender and distended, and bowel sounds were hyperactive. No masses were palpable. There was no significant medical history. His body temperature was 37°C. Laboratory findings showed a leukocyte count of 9700/mm^3^, whereas the hemoglobin and platelet values were 13 g/dl and 276,000/mm^3^, respectively. All other studies, including electrolytes and urinalysis, were within normal limits. Small intestine exhibited an air fluid level on the direct abdominal plain film. He was diagnosed with mechanical intestinal obstruction, and nasal decompression was performed. Emergency exploratory laparotomy was performed under general anesthesia. The distal part of the ileum was found to be markedly compressed by the mesodiverticular band within an area 60 cm proximal to the end of the ileum (Figure 1). Ileal loops were dilated at the superior part of the mechanical obstruction. Obstruction was caused by trapping of a bowel loop by a mesodiverticular band. After separating the mesodiverticular band from the mesenterium, the ileal loop was released from the diverticulum. Resection of the Meckel's diverticulum (Figure 2) and functional end-to-end anastomosis of the bowel were performed. The diverticulum was confirmed as Meckel's diverticulum by histological examination. The patient recovered without any complications and was discharged after five days of hospitalization.

## 3. Discussion

Meckel's diverticulum was originally described by Fabricius Hildanus in 1598. However, it is named after Johann Friedrich Meckel, who established its embryonic origin in 1809 [6]. Meckel's diverticulum is the most common congenital anomaly of the small intestine, with a prevalence of approximately 1–3%, and is a true diverticulum containing all layers of the bowel wall [2, 5–7]. The average length of a Meckel's diverticulum is 3 cm, with 90% ranging between 1 cm and 10 cm and the longest being 100 cm. This diverticulum is usually found within 100 cm of the ileocaecal valve on the antimesenteric border of the ileum. The mean distance from the ileocaecal valve seems to vary with age, and the average distance for children under 2 years of age is known to be 34 cm. For adults, the average distance of the Meckel's diverticulum from the ileocaecal valve is 67 cm [4]. Most cases that have Meckel's diverticulum are asymptomatic. Estimated risk for developing lifetime complications of the Meckel's diverticulum is around 4% [2, 5–7].

Most of the patients are asymptomatic, and the diagnosis is difficult to confirm preoperatively. Among the symptomatic patients, two types of heterotopic mucosa (gastric and pancreatic) are found histologically within the diverticula. The frequent complications of Meckel's diverticulum are hemorrhage, intestinal obstruction, and diverticulitis. Intestinal obstruction is the second most common complication of Meckel's diverticulum [3]. 

Elsayes et al. [1] noted that bowel obstruction accounts for up to 40% of symptomatic Meckel's diverticula. There are lots of mechanisms for bowel obstruction arising from a Meckel's diverticulum. Obstruction can be caused by trapping of a bowel loop by a mesodiverticular band, a volvulus of the diverticulum around a mesodiverticular band, and intussusception, as well as by an extension into a hernia sac (Littre's hernia) [1, 3]. Similarly, as in our case, obstruction can be caused by trapping of a bowel loop by a mesodiverticular band. The important aspect of our case is clear demonstration of the mesodiverticular band of a Meckel's diverticulum. According to our literature review, this is the most evident mesodiverticular band view published until now. 

Various imaging modalities have been used for diagnosing Meckel's diverticulum. Conventional radiographic examination is of limited value. Although of limited value, sonography has been used for the investigation of Meckel's diverticulum. High-resolution sonography usually shows a fluid-filled structure in the right lower quadrant having the appearance of a blind-ending, thick-walled loop of bowel. On computed tomography (CT), Meckel's diverticulum is difficult to distinguish from normal small bowel in uncomplicated cases. However, a blind-ending fluid or gas-filled structure in continuity with small bowel may be revealed. Abdominal CT is used for complicated cases such as intussusceptions. CT can help to confirm the presence of intussusception and distinguish between lead point and nonlead point intussusceptions [2, 8, 9]. 

Among the asymptomatic patients, whether all incidental Meckel's diverticula should be resected or not is an unresolved question. On the other hand, for the symptomatic patients, treatment should always include resection of the diverticulum or the segment of the bowel affected by the pathology [3, 10]. 

In conclusion, although Meckel's diverticulum is the most prevalent congenital abnormality of the gastrointestinal tract, it is often difficult to diagnose. The complications of Meckel's diverticulum should be kept in mind in the differential diagnosis of small bowel obstruction.
